# Orthoreovirus outer-fiber proteins are substrates for SUMO-conjugating enzyme Ubc9

**DOI:** 10.18632/oncotarget.12973

**Published:** 2016-10-28

**Authors:** Fei Yu, Hao Wang, Longlong Wang, Liqun Lu

**Affiliations:** ^1^ National Pathogen Collection Center for Aquatic Animals, Key Laboratory of Aquatic Genetic Resources of Ministry of Aquaculture, Shanghai Ocean University, Shanghai, PR China

**Keywords:** outer-fiber protein, reovirus, SUMOylation, Ubc9

## Abstract

Reoviruses are potential anticancer agents due to their ability to induce cell death in tumor cells. Grass carp reovirus (GCRV) is one of the best characterized models on reovirus pathogenesis *in vitro*. However, there is little known about how SUMOylation affects reovirus pathogenesis. The SUMO conjugating enzyme 9 (Ubc9) determines the targets of SUMOylation. Here, the protein interactions between reovirus outer fiber proteins, specifically GCRV-104 VP55, and Ubc9 were probed using a yeast two-hybrid system. The N-terminal coiled-coil domain of VP55, containing a single lysine residue, was responsible for the interaction between VP55 and Ubc9 in yeast. In solid phase binding assays, a single amino acid mutation (K87R) prevented Ubc9 from binding to VP55. Overexpression of Ubc9 enhanced GCRV-104 infection efficiency, and knockdown of Ubc9 in CIK cells inhibited viral replication, which suggested that Ubc9 was a proviral factor. Furthermore, Ubc9 was shown to bind outer fiber proteins from type II GCRV, avian reovirus and mammalian reovirus in yeast. To our knowledge, this is the first study to show that Ubc9 binds to reovirus outer-fiber proteins and likely contributes to efficient orthoreovirus replication. These results suggest that SUMOylation modifications could be targeted to improve the therapeutic efficacy of oncolytic reovirus.

## INTRODUCTION

*Rotavirus, Aquareovirus* and *Orthoreovirus* are three genera in the family *Reoviridae*, which is characterized by segmented double-stranded RNA (dsRNA) genome and non-enveloped virion of 20-sided icosahedral capsid comprised of an outer and inner protein shell [1–3]. Grass carp reovirus (GCRV) is an *aquareovirus* that causes hemorrhagic disease in grass carp (*Ctenopharyngon idellus*) and it is one of the most extensively characterized aquatic reoviruses due to its virulence *in vivo* and *in vitro* [4]. GCRV is typically categorized as a type C *aquareovirus* because it lacks an outer-fiber protein. However, two novel strains of GCRV, GCRV-HZ08 and GCRV-104 bearing an outer-fiber protein on the virion surface, were recently isolated from grass carp [5, 6] and classified as piscine orthoreoviruses [7].

Orthoreoviruses infect many mammalian species including mice, chimpanzees, dogs, cats, cattle, sheep, swine, horses, and monkeys [8]. Mammalian orthoreoviruses have not been linked to any serious disease in humans. In fact, the human reovirus Type 3 Dearing strain (ORV-T3D/MRV-T3D) has been evaluated as an anti-cancer agent in several clinical trials because it preferentially induces cell death in cancer cells [9, 10]. To date, multiple types of cancer have been shown to respond to MRV-T3D treatment in murine models [8]. The therapeutic effect of MRV is largely (but not exclusively) due to its replication in cells with dysfunctional signaling cascades leading to KRAS-overexpression and subsequent PKR inhibition [11, 12], and MRV also activates human dendritic cells to promote innate antitumor immunity [13].

GCRV-104 is unique in that it is a pathogenic piscine orthoreovirus strain and induces extensive cytopathic effects in host *Ctenopharyngodon idellus* kidney (CIK) cells [6]. This suggests that GCRV-104 is a good model system for studying orthoreovirus host-pathogen interactions. In an effort to screen interacting partners for the GCRV-104 outer-fiber protein (VP55) through yeast two-hybrid screen, grass carp Ubc9 was identified as one of the major candidate proteins interacting with VP55 in yeast [14]. Ubc9 is one of the central molecules regulating the process of SUMOylation, which plays an important role in multiple intracellular processes including protein subcellular localization, transcription, DNA repair, chromosome dynamics, and innate immunity [15]. SUMOylation is tightly regulated by a three-enzyme pathway containing an E1 (activating enzyme), E2 (conjugating enzyme), and E3 (ligase) component [16]; and Ubc9 is the sole SUMO E2 enzyme [17]. Exogenous expression of Ubc9 is known to enhance SUMOylation, while cells with Ubc9 knocked-down are SUMOylation deficient [15]. Interestingly, SUMOylation has been shown to negatively regulate the innate immune response by decreasing IFN production [18].

Several host and viral proteins are known to be SUMOylated during viral infection. SUMOylation is not restricted to host cell proteins and is exploited to create conditions favorable to infection by many viruses including dengue virus [19], white spot syndrome virus infection in *Fenneropenaeus chinensis* [20], influenza A virus [21], hepatitis C virus [22], herpes simplex virus 1 [23] and Kaposi's sarcoma-associated herpes virus (KSHV) [24]. However, interaction between key viral proteins of any orthoreovirus and SUMOylation system remains unknown. Therefore, this study aims to characterize the binding interactions between Ubc9 and GCRV-104 VP55 specifically, and also probes the association with other representative orthoreovirus outer fiber proteins in general.

## RESULTS

### Grass carp Ubc9 binds to the outer-fiber protein VP55 of GCRV-104

**Figure 1:**
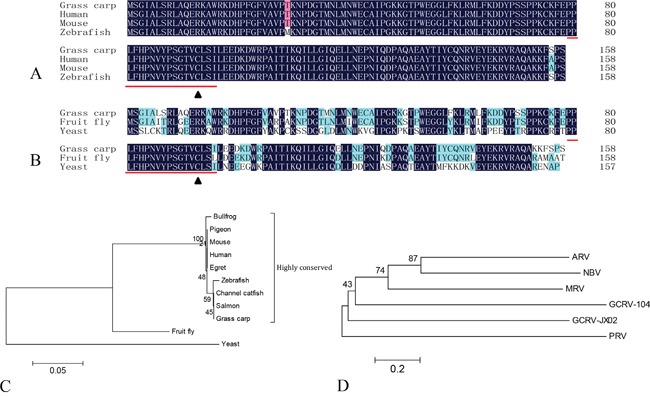
Phylogenetic analysis of grass carp Ubc9 and orthoreovirus fiber proteins Multiple sequence alignments are shown comparing the amino acid sequence of grass carp Ubc9 to higher order **A.** and lower order **B.** animal models. The conserved ubiqutin-conjugating catalytic (UBCc) domain is indicated with underlined text and the Cys93 residue is marked with a black triangle. Phylogenetic analysis of grass carp Ubc9 **C.** and orthoreovirus fiber proteins **D.** Phylogenetic trees were generated on the basis of the amino acid sequences. Species and accession numbers for Ubc9: Bullfrog (ACO52076.1); Pigeon (EMC86701.1); Mouse (NM_011665.4); Human (AAA86662.1); Egret (KFP20677.1); Zebrafish (AF128240.1); Channel catfish (NP_001187932); Salmon (ACI33971.1); Grass carp (KU760729); Fruit fly (AB017606.1); and Yeast (X81846.1). Accession numbers for each virus and fiber protein: Grass carp reovirus strain 104 (GCRV-104, AFG73678.1); Grass carp reovirus strain JX02 (GCRV-JX02, ALS05356.1); Piscine reovirus (PRV, KC915033.1); Mammalian orthoreovirus 3 Strain T3D (MRV-T3D, EF494441.1); Avian reovirus strain S1133 (ARV-S1133, AAK18188.1); and Nelson Bay orthoreovirus (NBV, AAF45159.1).

To better understand the interactions between orthoreovirus and the host SUMOylation system, we characterized the interaction between Ubc9 and VP55. Full-length cDNA of grass carp *Ubc9* was obtained by RT-PCR from the total mRNA extracted from CIK cells and directly submitted to GenBank (accession number: KU760729). The 477 bp open reading frame (ORF) of *Ubc9* gene was analyzed at both the nucleic acid and amino acid levels. The ORF encoded a putative 158 amino acid protein with a theoretical molecular weight of 18.02 kDa and an isoelectric point of 8.87. The grass carp Ubc9 protein contained an ubiquitin-conjugating catalytic (UBCc) domain and a Cys93 residue [25], which was evolutionarily conserved in other species (Figure 1A and 1B). In a phylogenetic analysis, grass carp Ubc9 clustered in the same branch with teleost Ubc9 proteins (Figure 1C). Grass carp Ubc9 shared nearly 100% similarity with the Ubc9 of either mammalian or zebrafish, and had less similarity with invertebrate animals.

**Figure 2:**
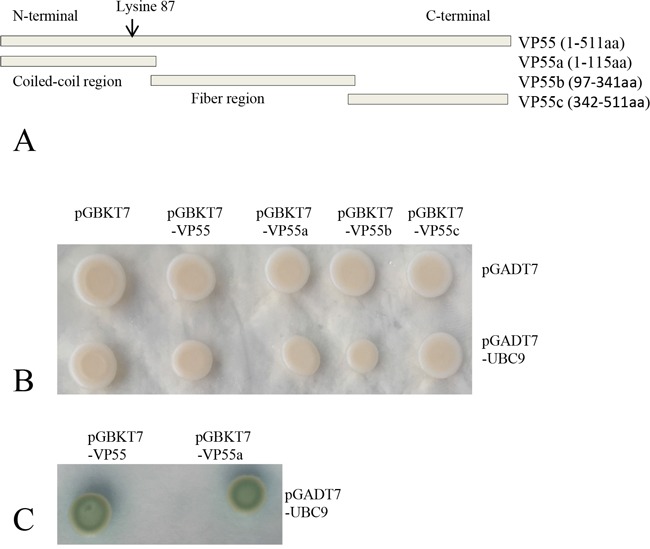
Ubc9 binds to full-length and truncated protein VP55 in yeast **A.** Schematic of the full-length and truncated VP55 proteins. The predicted coiled-coil domain and fiber protein regions are indicated. The full-length and three truncated fragments were inserted into the pGBKT7 vector. **B.** Yeast transformants containing the bait and prey plasmids were grown on SD/−Trp-Leu plates. **C.** Yeast transformants containing the bait and prey plasmids were grown on SD/−Trp-Leu-His-Ade/X-α-gal plates. Blue colonies indicate an interaction between the bait and prey (Only shown the positive colonies).

Yeast two-hybrid screening efficiently identified potential interaction partners for VP55 in our previous report [14]. Taking advantage of this system, we screened for domain(s) responsible for the association between VP55 and Ubc9. As shown in Figure 2A, VP55 contained an N-terminal α-helical coiled-coil domain (VP55a, 1-115 aa), middle fiber region (VP55b, 97-341 aa), and C-terminal region (VP55c, 342-511 aa). For the yeast two-hybrid Gal4 system, *VP55*a, b, and c were cloned to the bait vector separately and *Ubc9* ORF was cloned to the prey vector. The assays revealed that Ubc9 bound to full-length VP55 and the N-terminal coiled-coil domain of VP55, but not the middle fiber region or C-terminal region of VP55 (Figure 2B and 2C).

**Figure 3:**
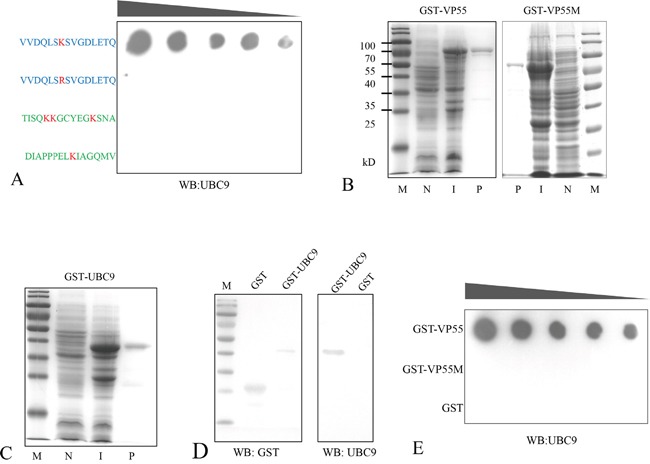
Lysine 87 of VP55 is involved in its interaction with Ubc9 **A.** Binding assay of synthesized polypeptides and Ubc9. The polypeptides containing Lys87 or the K87R mutation are shown in blue and negative control polypeptides from the C-terminal region of VP55 are shown in green. The polypeptides were immobilized on a solid phase, and incubated with GST-UBC9. Dots indicate Ubc9 binding and were probed with an anti-Ubc9 polyclonal rabbit antibody. **B** and **C.** GST-tagged proteins were induced and purified from *E. coli*. Representative images from an SDS-PAGE analysis of GST-VP55/GST-VP55M (K87R)/GST-Ubc9 are shown. Lanes: (N) non-induced, (I) induced, and (P) purified. **D.** A Western blot analysis of the purified proteins GST and GST-UBC9 probed with a monoclonal mouse antibody against GST and the anti-Ubc9 antibody. **E.** Representative image from an assay showing the interaction between VP55/VP55M and Ubc9. Purified GST-VP55, GST-VP55M or GST (from 0.25-10 μg) were immobilized on a solid phase substrate then incubated with GST-UBC9. Dots indicate Ubc9 binding and were probed with the anti-Ubc9 antibody.

The SUMO-interacting motif (SIM) has been shown to have a hydrophobic core (V/I-X-V/I-V/I) flanked by a cluster of negatively charged residues [26]. Ubc9 regulates SUMO target discrimination and can bind specifically to lysine residues in a nonconsensus SUMO modification motif [17, 27, 28]. There is a single residue in the VP55 coiled-coil domain at position 87 (Lys87), suggesting that Lys87 may be involved in Ubc9 binding. To confirm that Lys87 was the binding site for Ubc9, the *in vitro* interaction between Ubc9 and a polypeptide from the partial coiled-coil domain of VP55 (81-96 aa) encompassing Lys87 was assessed by solid-phase binding assay. Two polypeptides from the C-terminal region of VP55 (424-438 aa and 443-457 aa) were used as negative controls. The peptides were synthesized and GST tagged Ubc9 (Figure 3C and 3D) was prepared from *E. coli*. The binding assays revealed that the partial coiled-coil domain (81-96 aa) interacted with Ubc9, but 81-96 aa K87R did not (Figure 3A). To further confirm the result, the binding of a mutated VP55 with Lys87 removed (81-96aa K87R) to Ubc9 was also assessed. The VP55 and K87R VP55 mutant (Figure 3B) were also prepared from *E. coli*. In another dot-blot overlay assay, GST tagged Ubc9 was shown to interact with VP55, however, the VP55 mutant (K87R) lost the binding ability (Figure 3E). These results indicated that residues 81-96 were sufficient for VP55 binding and that Lys87 was necessary for the interaction.

### Expression levels of Ubc9 correlated with the replication efficiency of GCRV-104 in CIK cells

**Figure 4:**
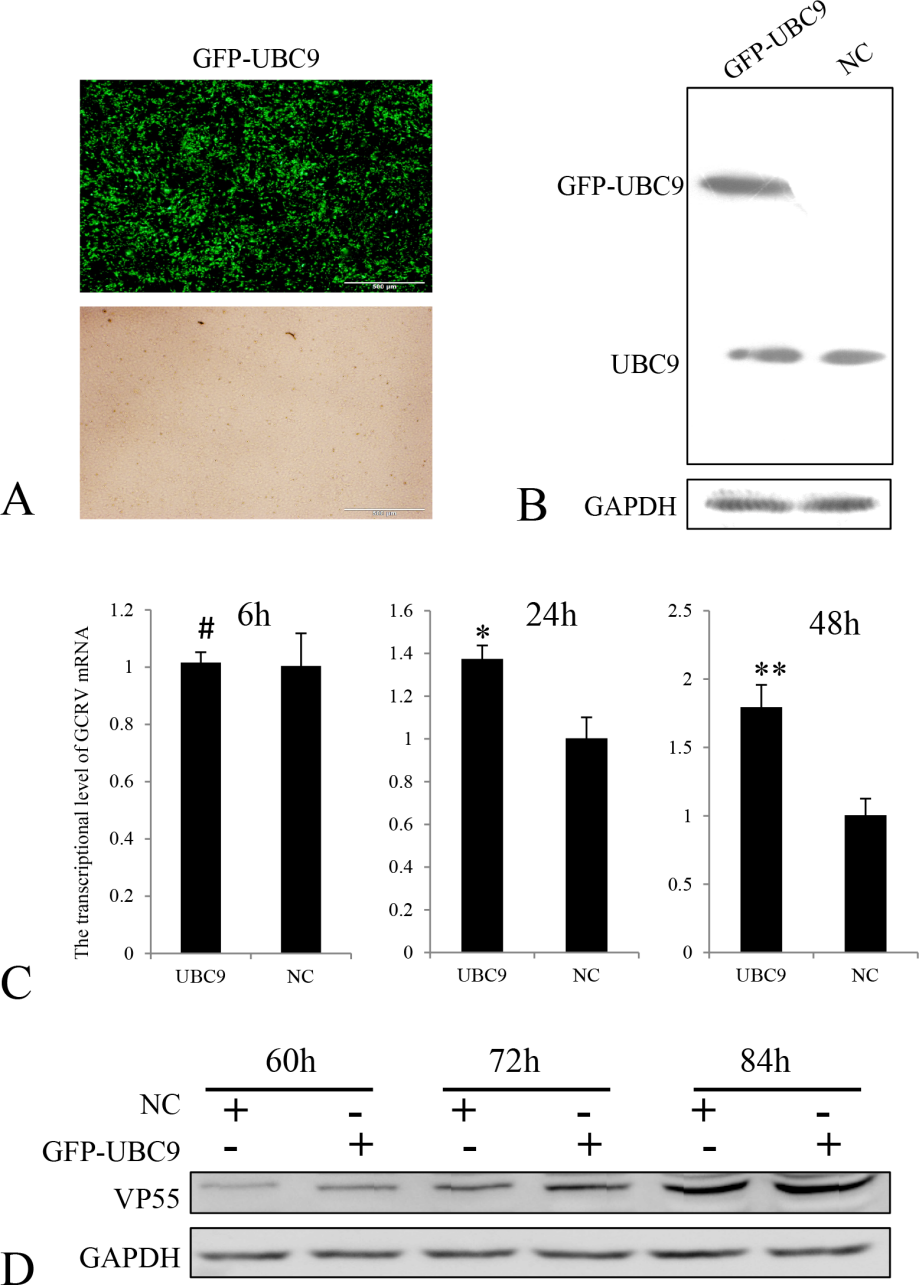
Overexpression of Ubc9 promoted GCRV replication in CIK cells **A.** Overexpression of GFP-UBC9 was confirmed by fluorescence microscopy (upper panel). The lower panel shows the same field under a visible light phase microscope. Scale bars = 500 μm. **B.** Ubc9 expression was measured in CIK cells transfected with pEGFP-UBC9 or mock cells were analyzed by Western blotting. Ubc9 expression was normalized to GAPDH expression. **C.** CIK cells were infected with GCRV-104 at a multiplicity of infection of 10. The levels of VP55 mRNA in Ubc9-overexpressing cells were analyzed by real time RT-PCR at the indicated time points and compared to VP55 expressed in mock infected cells (NC). Error bars indicate the standard deviation of the mean for experiments performed in triplicate. # no significant difference,*P < 0.05 and **P < 0.01. **D.** Expression of VP55 in cells was analyzed by Western blotting and normalized to GAPDH at the indicated time points.

Ubc9-expression level has been linked with infection efficiency for several other viruses [29]. Therefore, we evaluated the effects of overexpression of Ubc9 on GCRV-104 replication in CIK cells. Ubc9 was cloned into the eukaryotic expression vector pEGFP-N1, and transfected into CIK cells. Positive selection by G418 was used to identify cells expressing EGFP-Ubc9. More than 70% of CIK cells were found to overexpress EGFP-Ubc9 before the infection experiments were performed (Figure 4A and 4B). Similar to mammalian cells [30], immunofluorescence assays indicated that Ubc9 was primarily expressed in the nucleus (data not shown). The mRNA and protein expression levels of VP55 in cells overexpressing Ubc9 were monitored at different time points post infection (Figure 4C and 4D). Compared to cells that did not overexpress Ubc9, the Ubc9-overexpressing cells resulted in significantly higher expression levels of *VP55* mRNA at 24 h and 48 h post infection and VP55 protein at 60 h, 72 h and 84 h post infection.

**Figure 5:**
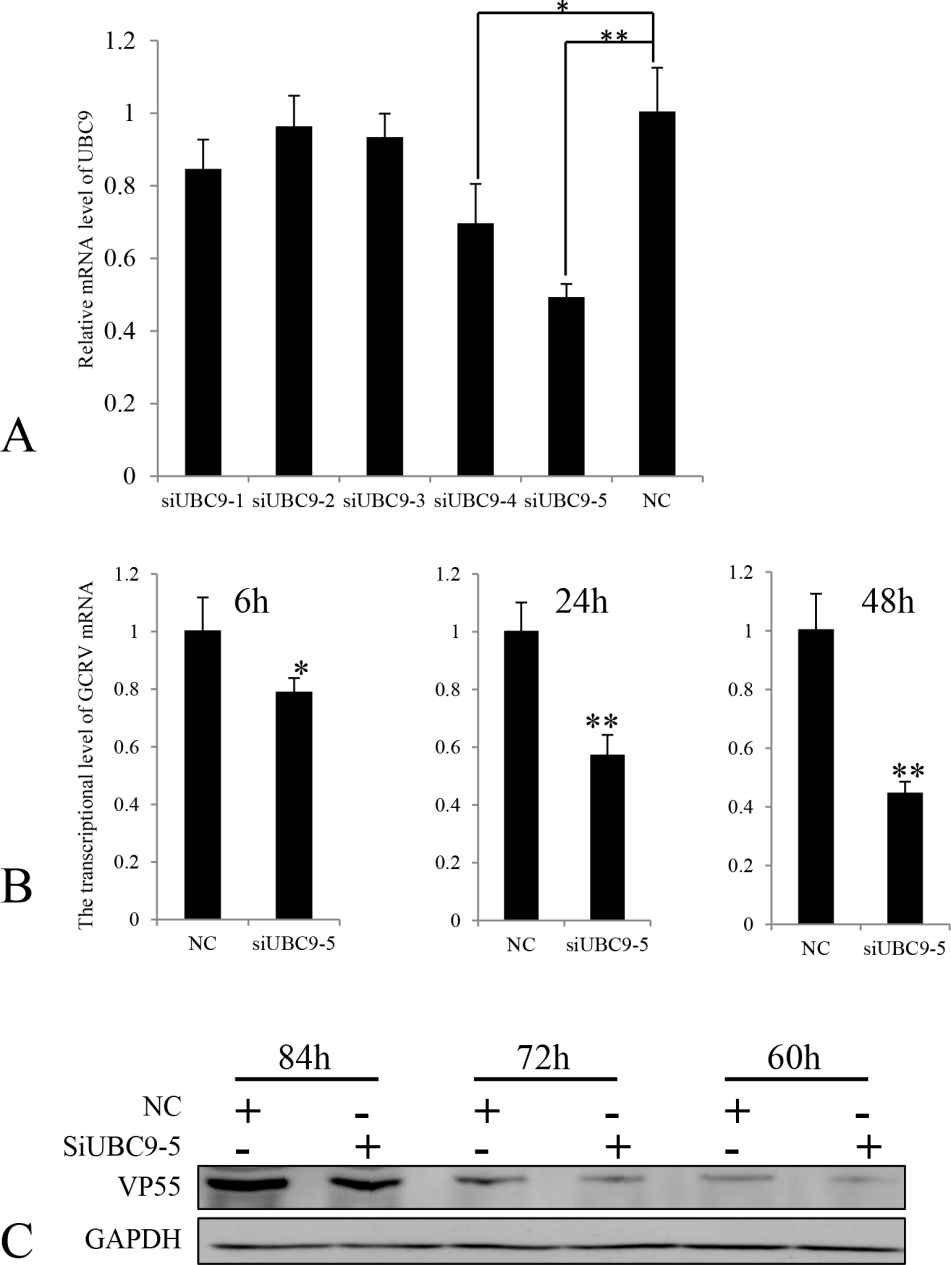
The expression of VP55 was reduced in the absence of Ubc9 **A.** Five different siRNAs targeting Ubc9 were transfected into CIK cells. The expression levels of *UBC9* mRNA were measured by real time RT-PCR and compared to Ubc9 expressed in CIK cells transfected with siRNA-NC (NC). Data is shown as the mean ± SE from experiments performed in triplicate. *P < 0.05 and **P < 0.01. **B.** CIK cells were infected with GCRV-104 at a multiplicity of infection of 10. The levels of VP55 mRNA in Ubc9-silenced cells were analyzed by real time RT-PCR at the indicated time points and compared to the expression in mock infected cells (NC). Error bars indicate the standard deviation of the mean for experiments performed in triplicate. *P < 0.05 and **P < 0.01. **C.** Expression of VP55 in cells with reduced Ubc9 expression were analyzed by Western blotting. The expressed signals were normalized to GAPDH levels at the corresponding time points.

To understand the effects of reduced expression of Ubc9 in CIK cells infected with GCRV-104, siRNA was used to inhibit *Ubc9* expression. Five siRNAs targeting the *Ubc9* gene, as well as the negative control siRNA (siRNA-NC), were synthesized and transfected into CIK cells. The expression levels of *Ubc9* mRNA were measured by real time RT-PCR. Our results revealed that siUbc9-5 was the most efficient inhibitor for *Ubc9* gene expression (Figure 5A). VP55 expression was measured in GCRV-infected cells post transfection with siUbc9-5 at different time points. Compared to control cells, the mRNA (Figure 5B) levels of *VP55* were significantly lower at 6 h, 24 h and 48 h post infection in cells with reduced expression of *Ubc9*, and consistently, the protein expression levels of VP55 were significantly lower at 60 h, 72 h and 84 h post infection (Figure 5C). Taken together, these results suggested that Ubc9 expression enhanced GCRV-104 replication.

### Binding of Ubc9 to outer capsid fiber protein is conserved for orthoreoviruses

**Figure 6:**
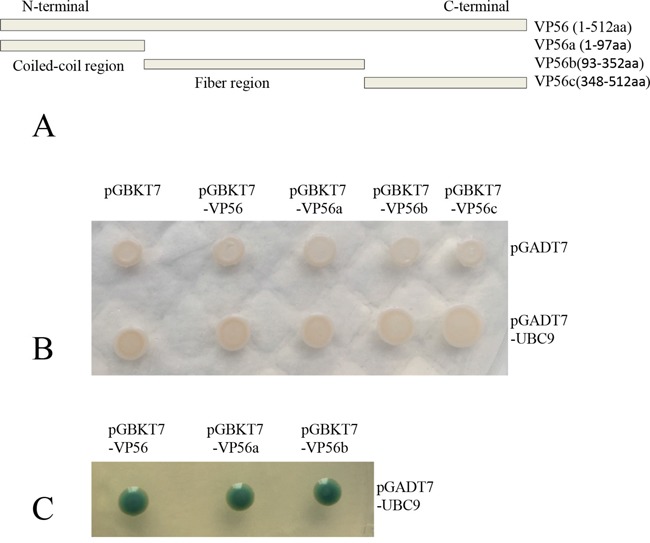
Ubc9 binds to the outer fiber protein from type II GCRV in yeast **A.** Schematic of full-length and truncated VP56. The predicted coiled-coil domain and fiber protein regions are indicated. The full-length and three truncated fragments were inserted into the pGBKT7 vector. **B.** Yeast transformants containing the bait and prey were grown on SD/−Trp-Leu plates. **C.** Yeast transformants containing the bait (VP56/VP56a/VP56b) and prey (UBC9) were grown on SD/−Trp-Leu-His-Ade/X-α-gal plates. Blue colonies indicate an interaction between the bait and prey (Only the positive colonies were shown).

Given the similarity between the VP55 of GCRV-104 and the outer fiber protein of GCRV-JX02, VP56, the interaction between VP56 and Ubc9 was probed by the well-established yeast two-hybrid system. Similar to VP55, VP56 consisted of three structural domains including the N-terminal α-helical coiled-coil domain (VP56a, 1-97 aa), middle fiber region (VP56b, 93-352 aa), and C-terminal region (VP56c, 348-512aa; Figure 6A). Each of the domains of VP56 was expressed from the bait vector, and Ubc9 was expressed from the prey plasmid in yeast. Our analysis demonstrated that Ubc9 bound to the full-length protein, the coiled-coil domain, and the middle fiber region of VP56, but not the C-terminal region (Figure 6B and 6C). These data suggested that VP56 interacted with Ubc9 and might have two or more different interaction sites.

**Figure 7:**
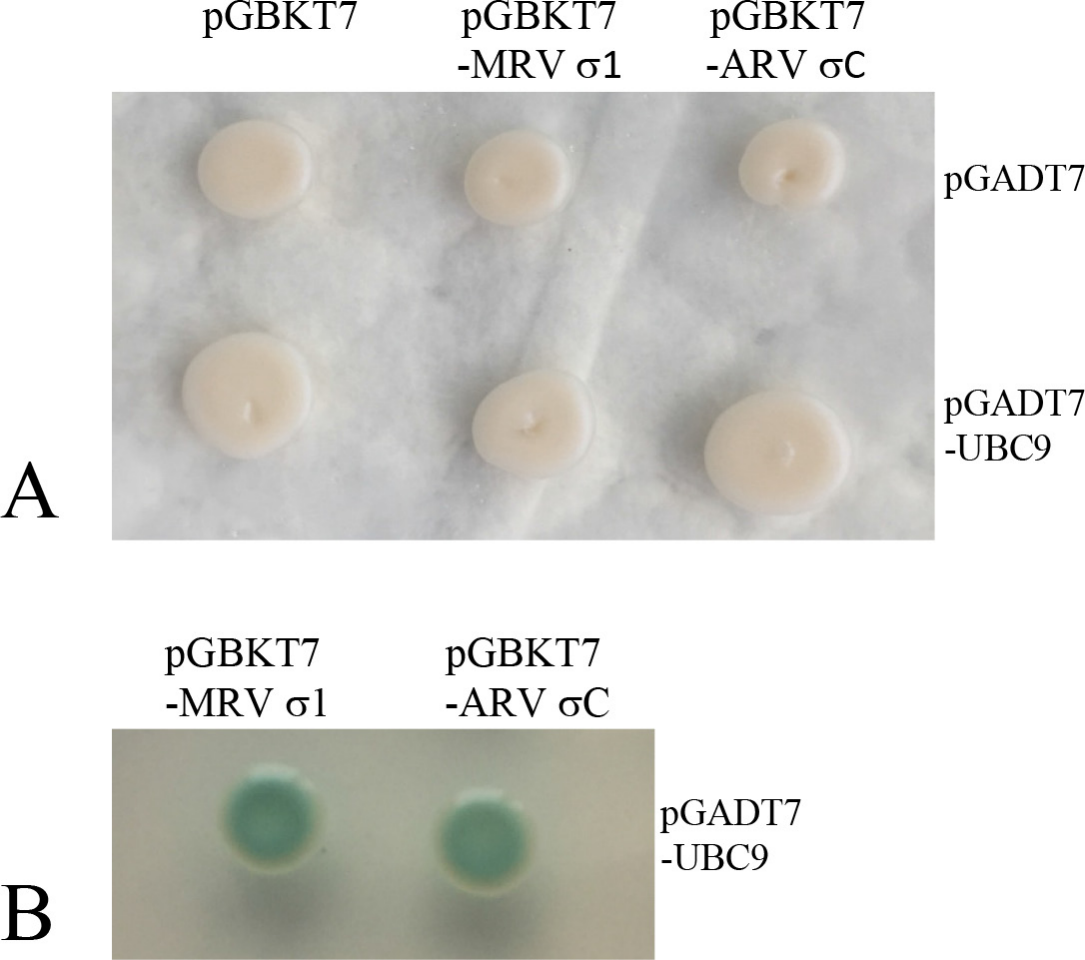
Ubc9 binds outer fiber protein of avian or mammalian orthoreovirus in yeast **A.** Yeast transformants containing the bait and prey were grown on SD/−Trp-Leu plates. **B.** Yeast transformants containing the bait (MRV σ1/ARV σC and prey (UBC9) were grown on SD/−Trp-Leu-His-Ade/X-α-gal plates. Blue colonies indicate an interaction between the bait and prey (Only the positive colonies were shown).

Representative strains of orthoreoviruses containing fiber proteins were listed in Table 1 and the deduced protein length (aa), pI values of the fiber proteins were considerably different. Phylogenetic analyses indicated that the nearest phylogenetic distance was not between VP55 and VP56, and the fiber proteins tended to share low identities with each other (Table 1 and Figure 1D). We next investigated the interactions between Ubc9 and fiber proteins encoding by other representative orthoreoviruses. In yeast two-hybrid assays, the fiber proteins of avian reovirus (ARV σC) and mammalian reovirus (MRV σ1) were shown to bind to Ubc9 (Figure 7). The result indicated that Ubc9 binding to outer capsid fiber proteins was conserved in orthoreoviruses.

**Table 1:** Deduced length (aa), pI values and pairwise evolutionary distances of orthoreovirus fiber proteins

Representative strains^**^	Segerment/aa/pI	Main host	Estimates of evolutionary divergence between protein sequences^*^					
			GCRV-104	GCRV-JX02	PRV	MRV	ARV	NBV
GCRV-104	7/511/5.5	Grass carp		2.10	2.38	2.13	1.97	2.02
GCRV-JX02	7/512/5.3	Grass carp			2.18	1.93	2.10	2.02
PRV	10/315/5.9	Atlantic salmon				2.07	2.21	2.24
MRV-T3D	7/455/5.3	Human					1.51	1.70
ARV-S1133	7/326/4.9	Fowl						1.37
NBV	7/323/6.9	Flying fox						

* Pairwise evolutionary distances between protein sequences were calculated by MEGA version 5.1.

** Grass carp reovirus strain 104 (GCRV-104, AFG73678.1); Grass carp reovirus strain JX02 (GCRV-JX02, ALS05356.1); Piscine reovirus (PRV, KC915033.1); Mammalian orthoreovirus 3 Strain T3D (MRV-T3D, EF494441.1); Avian reovirus strain S1133 (ARV-S1133, AAK18188.1); Nelson Bay orthoreovirus (NBV, AAF45159.1).

## DISCUSSION

In this study, we provided evidence that the GCRV-104 VP55 protein interacted with Ubc9 at Lys87 in the N terminus. To our knowledge, we were the first to show an interaction between the SUMOylation system and an orthoreovirus outer fiber protein. Overexpression of Ubc9 promoted GCRV-104 replication and knockdown of Ubc9 resulted in reduced infection efficiency. Furthermore, we provided evidence that Ubc9 bound to outer fiber proteins from type II GCRV, avian and mammalian orthoreoviruses, suggesting they might also be potential targets for SUMOylation, regardless of viral host range (Teleost, Avian or Mammal; Table 1). We have demonstrated that the interaction between outer-fiber protein and SUMOylation system likely favored GCRV-104 replication, suggesting that orthoreoviruses might utilize the SUMOylation machinery for efficient viral replication. However, this observation should be analyzed by direct SUMOylation assay in future studies.

The implications of interactions between the SUMOylation machinery and orthoreoviruses remained to be defined. The therapeutic oncolytic effects of orthoreoviruses might be enhanced through targeted manipulations of the SUMOylation status of key structural proteins. For example, in animal models treatment with MRV-T3D is beneficial for many different cancers [31]. However, the utility of MRV-T3D may be limited by the absence or inaccessibility of the JAMA-1 receptor [32, 33]. Given that the reovirus outer-fiber proteins are used as a viral attachment protein [34], SUMO modification of these proteins may regulate receptor binding and could potentially be used to increase tropism for host tumor cells. Clarifying the role of SUMO modification in orthoreovirus infection might also contribute to addressing safety concerns around using orthoreoviruses as anti-cancer immunotherapy agents.

Besides fiber proteins, SUMOylation might also affect the other structure or non-structure proteins in orthoreoviruses. In rotaviruses, SUMO can be covalently conjugated to the viroplasm proteins VP1, VP2, NSP2, VP6, and NSP5 [35]. In addition, SUMO-modification of cytomegalovirus (CMV) IE1 and IE2 proteins of Cytomegalovirus (CMV) is important for their activity regulating early events in the virus infection cycle [36]. The involvement of other reoviral proteins that may modify and/or be modified by the SUMOylation system was beyond the scope of this study. In addition, SUMOylation may affect the stability of VP55, which should be an area for future research. While there are many unanswered questions, our results support a role for the SUMOylation pathway in orthoreovirus infection and may contribute to the development of anti-cancer agents based on orthoreoviruses.

## MATERIALS AND METHODS

### Yeast, virus and cell culture

*Saccharomyces cerevisiae* stain AH109 was from Clontech and preserved in the National Pathogen Collection Center for Aquatic Animals. The GCRV-104 isolate was obtained from the Yangtze River Fisheries Research Institute, Chinese Academy of Fishery Sciences, and the GCRV-JX02 isolate was isolated by our lab [37]. *Ctenopharyngodon idellus* kidney (CIK) cells were obtained from China Center for Type Culture Collection (Wuhan, China) and passaged in our laboratory for fewer than 6 months after resuscitation. CIK cells were grown in MEM medium containing 10% fetal bovine serum. Viruses used in this study were collected from the infection supernatant in CIK cells and the virus titer was determined as previously described [38].

### Cloning and sequence analysis of grass carp Ubc9

We have previously published a cDNA library constructed from grass carp mRNA that was sequenced using Illumina Hiseq 2000 technology [39]. The full-length Ubc9 sequence was obtained from the cDNA sequences using local blasting in the BioEdit software program. Total RNA was extracted from CIK cells using TRIzol (Invitrogen, USA) according to the manufacturer's protocol. Primers (Table 2) were designed to amplify the Ubc9 ORF by RT-PCR (cDNA Synthesis Kit, TaKaRa, Japan). A 477-bp fragment of the Ubc9 gene was cloned into the pMDTM19-T Vector (Takara, Japan) using TA cloning. The cDNA sequence and putative amino acid sequence similarity analyses were performed by blasting the Genbank database (http://www.ncbi.nlm.nih.gov/). Bioinformatics analyses were performed using the ExPASy Resource Portal (http://www.expasy.org/). Multiple sequence alignments for the amino acid sequences were performed using the DNAman software program. Based on the amino acid sequences of Ubc9 from the indicated species (Figure 1), MEGA version 5.1 was used to generate a phylogenetic tree utilizing the neighbor-joining algorithm and to compute the estimates of evolutionary divergence between amino acid sequences [40].

**Table 2:** Nucleotide sequences used in this study

Genes	Sense (5′-3′)	Antisense (5′-3′)	Recognition sites	Application
UBC9	ATGTCTGGCATTGCT CTGAGTCGAC	TTACGACGGGGAGA ATTTTTTGGCC		Full length
UBC9	GGAATTC*CATATG*ATGTCTG GCATTGCTCTGAGTC	GCG*GGATCC*CGACGGGGA GAATTTTTTGG	Nde I/EcoR I	pGADT7-UBC9
VP55	CCG*GAATTC*ATGGA CGATCAAGCGCTCG	GCG*GGATCC*CGCAAGTGACA GGCCGCCA	EcoR I/BamH I	pGADT7-VP55
UBC9	GGAATTC*CATATG*ATG TCTGGCATTGCT	AGG*GGGCCC*CCGTACGAC GGGGAGAATT	Nde I/Sma I	pGBKT7-UBC9
VP55	CCG*GAATTC*ATGGACGAT CAAGCGCTCGCA	CGC*GGATCC*GGCAAGTG ACAGGCCGCCACC	EcoR I/BamH I	pGBKT7-VP55
VP55a	GGAATTC*CATATG*ATGGACGAT CAAGCGCTCGCAAAC	CCG*GAATTC*GACTAATGGTGG ATTATACTCATT	Nde I/EcoR I	pGBKT7-VP55a
VP55b	GGAATTC*CATATG*ATGGATGGT GTTGAGAGTGAGTTAG	CCG*GAATTC*CGGAGATACGTC CAACGACAATG	Nde I/EcoR I	pGBKT7-VP55b
VP55c	CATG*CCATGG*CAATGCGCGACT TCTCTATCACCACAGG	CCG*GAATTC*AGTGACAGGCCG CCACCATGACATC	Nco I/EcoR I	pGBKT7-VP55c
VP56	CATG*CCATGG*AGATGGCC ACTCGTGACAG	CGC*GGATCC*GGTACTTACA GCAAACTACCGT	Nco I/BamH I	pGBKT7-VP56
VP56a	GGAATTC*CATATG*ATGGCC ACTCGTGACAGCCG	CCG*GAATTC*ATCCACTAT GCCAGCCAAG	Nde I/EcoR I	pGBKT7-VP56a
VP56b	CATG*CCATGG*CAATGGCTGGC ATAGTGGATGCGAC	CCG*GAATTC*AGTCTTCTCC AAGCTTAAAT	Nco I/EcoR I	pGBKT7-VP56b
VP56c	CATG*CCATGG*CAATGAGCTTGGA GAAGACTCTTAAC	AAAA*CTGCAG*CCTTACA GCAAACTACCGTCC	Nco I/Pst I	pGBKT7-VP56c
σC	*GAATTC*ATGGCGGGT CTCAATCCATC	*GTCGAC*TTAGGTGTC GATGCCGGTACGC	EcoR I/Sal I	pGBKT7-ARV (C
σ1	*GAATTC*ATGGATCCTCGCC TACGTGAAG	*GTCGAC*TCACGTGAA ACTACGCGGGTAC	EcoR I/Sal I	pGBKT7-MRV σ1
UBC9	CCG*GAATTC*CATGTCTGG CATTGCTCTGAG	ATAAGAAT*GCGGCCGC*T TTACGACGGGGAGAATTTT	EcoR I/Not I	pGEX-4T-3-UBC9
VP55	CCG*GAATTC*CATGGACGATCAA GCGCTCGC	TT*GCGGCCGC*AGCAAGTG ACAGGCCGCCAC	EcoR I/Not I	pGEX-4T-3-VP55
VP55M	GTGGATCAACTGTCTCGATCTG TTGGTGATCTG	CAGATCACCAACAGATCG AGACAGTTGATCCAC		pGEX-4T-3-VP55M
UBC9	CCC*AAGCTT*ATGTCTGGCATT GCTCTGAG	CCG*GAATTC*CGTACGACGGG GAGAATTTT	Hind III/EcoR I	pEGFP-N1-UBC9
18S	ATTTCCGACACGGAGAGG	CATGGGTTTAGGATACGCTC		Real time RT-PCR
VP55	ATCGTCTTCAACCGCATAG	GGGCGTTACTTCCCTCAAC		Real time RT-PCR
UBC9	TTATGAACTGGGAATGTGC	CTTTGGAGGTGATGAGGG		Real time RT-PCR
siUBC9-1	GGAGGAAAGACCAUCCAUUTT	AAUGGAUGGUCU UUCCUCCTT		Knockdown
siUBC9-2	CCCUGACGGUACCAUGAAUTT	AUUCAUGGUACC GUCAGGGTT		Knockdown
siUBC9-3	GGGAAGGAGGUCUGUUUAATT	UUAAACAGACCU CCUUCCCTT		Knockdown
siUBC9-4	GCUCCUAAAUGAACCGAACTT	GUUCGGUUCAUUU AGGAGCTT		Knockdown
siUBC9-5	GCACAAGAGCGCAAAGCAUTT	AUGCUUUGCGCUC UUGUGCTT		Knockdown
siUBC9-NC	UUCUCCGAACGUGUCACGUTT	ACGUGACACGUUC GGAGAATT		Knockdown

Note: Mutant site is marked with underlined text and restriction enzyme recognition sites are shown as italicized text.

### Plasmid construction

All primers and restriction enzyme recognition sites utilized to construct the plasmids were listed in Table 2. The coding regions of the VP55 and VP56 genes were amplified by RT-PCR from infected grass carp cells infected with GCRV-104 or GCRV-JX02, respectively. The Ubc9 ORF was amplified by RT-PCR from CIK cells. The bait vector pGBKT7 and prey vector pGADT7 (Clontech) were used in a yeast two-hybrid Gal4 screening system. The coding region of Ubc9 was inserted into pGADT7 (pGADT7-UBC9) and full-length VP55 or VP56 were inserted into pGBKT7 (pGBKT7-VP55, pGBKT7-VP56). Three truncated sequences of VP55 were also cloned into pGBKT7: (1) N-terminal α-helical coiled-coil domain (1-115 aa, pGBKT7-VP55a); (2) middle fiber region (97-341 aa, pGBKT7-VP55b); and (3) C-terminal region (342-511 aa, pGBKT7-VP55c). VP56 was also truncated into three parts: (1) N-terminal α-helical coiled-coil domain (1-97 aa, pGBKT7-VP56a); (2) middle fiber region (93-352 aa, pGBKT7-VP56b); and (3) C-terminal region (348-512 aa, pGBKT7-VP56c). In addition, ORF encoded by avian reovirus (pGBKT7-ARV σC) and mammalian reovirus (pGBKT7-MRV σ1) fiber protein genes (synthesized by ShangGong, China) were cloned into the bait vector. To obtain the GST-tagged recombinant proteins, the genes for Ubc9 and VP55 were cloned into the pGEX-4T-3 vector (pGEX-UBC9, pGEX-VP55). Lys87 was mutated to arginine in the pGEX-VP55 plasmid (K87R, pGEX-VP55M) using the Fast Site-Directed Mutagenesis Kit (TIANGEN, China). To create the GFP-tagged Ubc9, the Ubc9 fragment was ligated into the pEGFP-N1 vector (pEGFP-UBC9), which fused GFP to the C-terminus of Ubc9.

The plasmids were constructed following the manufacturer's protocol using restriction enzymes and T4-ligase enzyme (TaKaRa, Japan). All DNA fragments were purified with the Wizard SV Gel and PCR Clean-Up System (Promega, USA). All plasmids were sequenced by ShangGong (Shangahi, China) to ensure the sequence was correct.

### Yeast two-hybrid assay

The bait and prey plasmids were concurrently transformed into yeast (AH109) using the Yeastmaker™ Yeast Transformation System 2 (Clontech, USA). The yeast transformants were grown in triplicate on deficient medium plates SD/−Trp-Leu or SD/−Trp-Leu-His-Ade/X-α-gal (Clontech, USA) for 3-7 d at 30°C. Empty bait or prey plasmids and the irrelevant plasmids pGBKT7-NS26 or pGADT7-CiLITAF [41] were used for the negative control and positive control, respectively (Supplementary Figure S1). The assays to detect interactions between Ubc9 and the outer fiber proteins from avian reovirus (ARV σC) and mammalian reovirus (MRV σ1) were performed as above.

### Protein expression and purification and antiserum preparation

To obtain purified recombinant proteins, BL21 (DE3) *E. coli* transformed with pGEX-UBC9, pGEX-VP55, or pGEX-VP55M were cultivated in Luria-Bertani medium containing 100 μg ampicillin/mL at 37°C until the optical density reached 0.4-0.6 at 600 nm. Expression of the GST fusion proteins was induced using 0.1-0.5 mM IPTG (isopropyl-β-D-thiogalactopyranoside) for 4-6 h. The pellet was collected by centrifugation at 5000× g for 5 min and resuspended in PBS (140 mM NaCl, 2.5 mM KCl, 10 mM Na_2_HPO_4_ and 2 mM KH_2_PO_4_). The expressed protein was purified as previously described [42] or using the Glutathione Sepharose™ 4B kit instructions (GE Healthcare). Purified GST-VP55 was used to prepare polyclonal antiserum. Three New Zealand rabbits were intracutaneously immunized with 250 μg of GST-VP55 in Freud's adjuvant (Sigma, USA) at 2 week intervals for three times. Immunoglobulin (IgG) specific for VP55 was purified using Protein A Agarose (Santa Cruz).

### Dot-blot overlay assay and western blotting

The dot-blot overlay assay was performed as described previously [43]. Briefly, the protein samples (ranging from 0.25-10 μg/μl) were blotted onto 0.45 μm polyvinylidene fluoride (PVDF) membrane using a dot-blot apparatus (Bio-Rad, USA). The membrane was incubated with GST-Ubc9 diluted in PBST (140 mM NaCl, 2.5 mM KCl, 10 mM Na2HPO4, 2 mM KH2PO4 and 0.1% Tween) and then blocked for 2 h at 30°C in 5% fat-free milk dissolved in PBST. The primary (anti-UBC9, 1:3000, Santa Cruz) and secondary antibodies (HRP conjugated anti-rabbit IgG, 1:4000, Abcam) were diluted in PBST containing 2.5% fat-free milk. The polypeptides (ranging from 0.5-20 μg/μl) were blotted onto 0.22 μm PVDF and probed as described above. For Western blots, the protein samples were resolved by 10% or 12% SDS-PAGE and then transferred onto 0.45 μm PVDF membrane. The membranes were blocked for 2 h at 30°C in 5% non-fat milk dissolved in PBST. Expression of GAPDH (anti-GAPDH, Abclonal) was used as an internal control. The primary (anti-VP55, 1:4000, prepared; anti-GST, 1:4000, Abcam) and secondary antibodies (HRP conjugated anti-rabbit/mouse IgG, 1:4000, Abcam) were used to probe the Western blots. Bands were visualized using chemiluminescence (ECL) or a DAB Horseradish Peroxidase Color Deveiopment Kit (Beyotime, China).

### Overexpression or knockdown of Ubc9 gene expression prior to GCRV infection

To overexpress Ubc9, 10 μg of the pEGFP-Ubc9 plasmid were transfected into 80% confluent CIK cells using the Lipofectamine 3000 reagent (Invitrigen) in a 25 mm culture flask. The transfected CIK cells were grown and passaged in MEM medium containing 400 μg/mL G418 (Sigma Aldrich) to improve the ratio of cells overexpressing EGFP-Ubc9. The expression of EGFP-Ubc9 was examined by fluorescence microscopy (Olympus, Japan). The cells overexpressing EGFP-Ubc9 cells were grown in a 6-well plate for 24 h (8×10^5^ cells/well) and then incubated with GCRV-104 at a multiplicity of infection (MOI) of 10 for 1 h. The infection media was replaced with fresh medium.

To inhibit Ubc9 expression, 100 pmol of each siRNA targeting Ubc9 (shown in Table 2 (synthesized by GenePharma, China) was transfected into CIK cells in a 6-well plate (8×10^5^ cells) using the Lipofectamine 3000 reagent. Ubc9 expression levels were measured by real time RT-PCR and normalized to 18S rRNA levels. The CIK cells with inhibited Ubc9 expression were infected with GCRV-104 in a 6-well plate (8×10^5^ cells) as described above.

The infections were performed in triplicate and collected 6, 12, 24, 48, 60, 72 and 84 h post infection. Mock infected CIK cells served as the control.

### Real time RT-PCR

All of the primers used for real time RT-PCR were listed in Table 2. Total RNA was extracted from cells (8×10^5^ cells) using 500 μL of TRIzol (Invitrigen, USA). Two hundred nanograms (200 ng) of isolated RNA were reverse transcribed into cDNA (RT Master Mix Kit, TaKaRa, Japan). The expression levels of Ubc9 and VP55 in CIK cells were measured in triplicated using real time RT-PCR and normalized to the 18S rRNA level [44]. Real time RT-PCR was performed following the SYBR^®^ Premix Ex Taq II (TaKaRa, Japan) kit instructions using a CFX96 real-time PCR system (Bio-rad, USA). The relative expression levels of Ubc9 were determined in comparison to Ubc9-expressing cells transformed with siRNA-NC using the 2^−ΔΔCT^ method. The relative expression levels of VP55 were determined by being compared to mock infected cells. Data analysis was performed using SPASS software and a P<0.05 was considered statistically significant.

## SUPPLEMENTARY FIGURES


