# Supplementary material for: Orthoreovirus outer-fiber proteins are substrates for SUMO-conjugating enzyme Ubc9
Source: Oncotarget. 2016 Oct 28;7(48):79814–27. doi: 10.18632/oncotarget.12973 (PMC5346753; doi:10.18632/oncotarget.12973)
Supplement: Supplementary file 1 [file oncotarget-07-79814-s001.pdf]

Orthoreovirus outer-fiber proteins are substrates for SUMO-conjugating enzyme Ubc9

SUPPLEMENTARY FIGURES

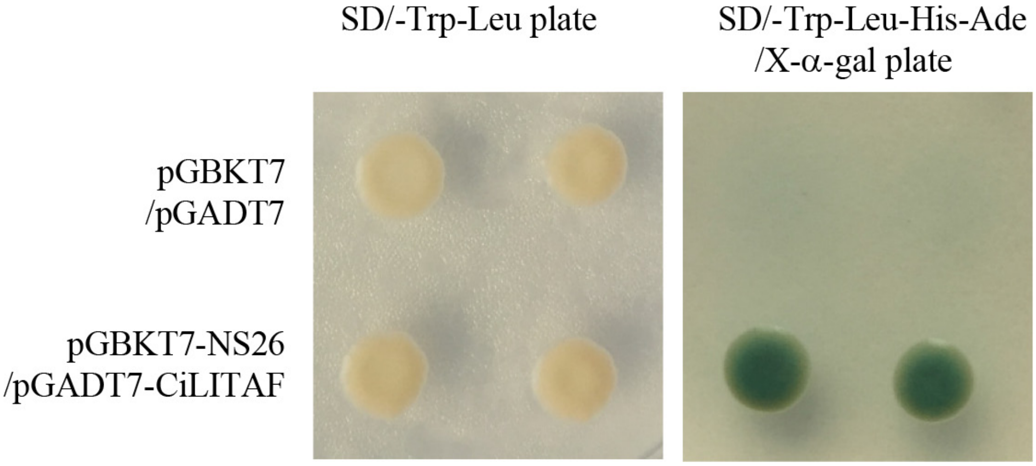

Supplementary Figure S1: The negative and positive controls in yeast, related Figure 2, 6 and 7. Empty bait/prey plasmids and the irrelevant plasmids pGBKT7-NS26/pGADT7-CiLITAF were used as the negative and positive controls, and the yeast transformants (two representative colonies) were grown on SD/-Trp-Leu plates (Left) and SD/-Trp-Leu-His-Ade/X-α-gal plates (Right).
